# Dengue, West Nile virus, chikungunya, Zika—and now Mayaro?

**DOI:** 10.1371/journal.pntd.0005462

**Published:** 2017-08-31

**Authors:** Peter J. Hotez, Kristy O. Murray

**Affiliations:** 1 Department of Pediatrics, Baylor College of Medicine, Houston, Texas, United States of America; 2 Department of Molecular Virology and Microbiology, National School of Tropical Medicine, Baylor College of Medicine, Houston, Texas, United States of America; 3 Texas Children’s Hospital Center for Vaccine Development, National School of Tropical Medicine, Baylor College of Medicine, Houston, Texas, United States of America; 4 Department of Biology, Baylor University, Waco, Texas, United States of America; 5 James A Baker III Institute for Public Policy, Rice University, Houston, Texas, United States of America; 6 Scowcroft Institute of International Affairs, The Bush School of Government and Public Service, Texas A&M University, College Station, Texas, United States of America; 7 Center for Vector-Borne and Zoonotic Diseases, National School of Tropical Medicine, Baylor College of Medicine, Houston, Texas, United States of America; Duke-NUS GMS, SINGAPORE

Is Mayaro virus infection the latest in a series of new arbovirus diseases expanding across the Western Hemisphere?

Since the launch of the 2000 Millennium Development Goals, we have seen an explosion of new arboviruses affecting the Americas. According to the Global Burden of Disease Study 2015, there has been a 143.1% increase in dengue fever cases between 2005 and 2015 [1], while between 1990 and 2013, dengue incidence in the Caribbean and tropical regions of Latin America increased severalfold [2]. In 1999, West Nile virus (WNV) was discovered in New York City, and by 2004, it had reached the West Coast [3]. Since its introduction, WNV has clinically affected more than 41,000 people, causing more than 1,700 deaths in the United States alone [4]. Then, in 2013–2014, both chikungunya virus and Zika virus infections emerged in the Americas, with both viruses rapidly spreading to dozens of countries over the course of 1 year. Both viruses have now affected millions of people, resulting in widespread morbidity [5, 6]. The factors responsible for the rapid expansion of arboviruses in the Western Hemisphere are still under investigation, but they likely include some of the new “Anthropocene” forces of climate change, deforestation, economic downturns and poverty, and the changing patterns of human migrations and urbanization [7].

But it does not look like the emergence and rapid expansion of new human arbovirus infections will abate anytime soon. In addition to Bourbon, Cache Valley, chikungunya, Heartland, Itaqui, Oropouche, Powassan, and Zika viruses [5], one of the latest to cause concern is Mayaro virus infection.

**Fig 1:**
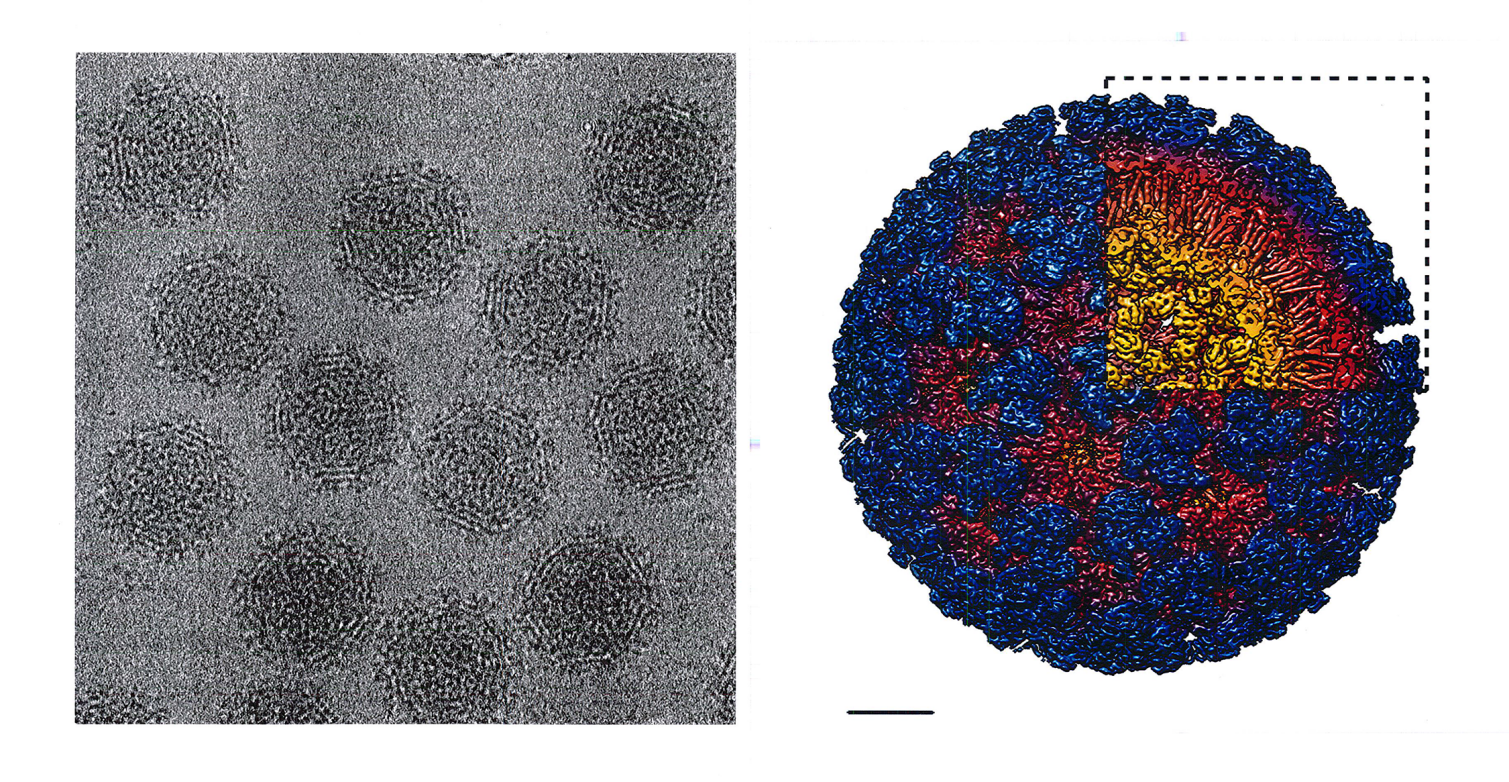
(Left) Cryoelectron micrograph of Mayaro virus strain 12A, (right) the 3D structure: View from outside the particle, with a cutaway only in 1 quadrant of the particle. Scale bar = 10 nm. *Courtesy of Drs. Jason Kaelber and A. Jonathan Auguste*.

Like chikungunya virus, Mayaro virus is an alphavirus and a member of the Togaviridae family of enveloped RNA viruses (Fig 1) [5, 6]. Mayaro virus was first isolated by Charles Anderson and his colleagues during the 1950s from humans with febrile illnesses in Trinidad and later characterized as an alphavirus by Jordi Casals and L. Whitman [8,9]. Subsequent outbreaks were reported from Bolivia and Brazil [10].

Mayaro virus has since been identified in the Amazon and other tropical regions of South America, where it has been mostly transmitted by *Haemogogus* mosquitoes and likely involves forest-dwelling nonhuman primates and possibly migratory birds as animal reservoirs [11]. However, the urban mosquito *Aedes aegypti* has now been also shown to be an experimental vector for Mayaro virus [12], so there is concern that just as yellow fever virus can exhibit a sylvatic (jungle) enzootic cycle to become urbanized and utilize *Aedes* mosquito species, Mayaro virus could follow a similar path [13]. However, it is still unknown whether *Ae*. *aegypti* or other *Aedes* mosquitoes are efficient vectors for Mayaro virus. In addition, there is a single report of Mayaro virus isolation from birds [14], but the role of birds in virus transmission remains unstudied.

Human Mayaro virus infection produces a constellation of symptoms that closely resemble the alphavirus infection caused by chikungunya, including fever, rash, and severe and prolonged arthralgias [6, 15]. One of the largest outbreaks of Mayaro virus was reported in 2015 by Scott Weaver’s group in a rural village located in northwestern Venezuela, where 77 cases were reported, including 19 individuals confirmed as seropositive [16]. Such findings together with the first report of Mayaro virus infection in an HIV-infected patient [17] prompted concerns that Mayaro virus could become an important emerging pathogen in South America [16], leading to early attempts to develop attenuated or other vaccines [18]. However, it remains unclear if there have been any significant ecological changes associated with the Venezuelan outbreak or whether the findings reflect improvements in pathogen surveillance technologies.

**Fig 2:**
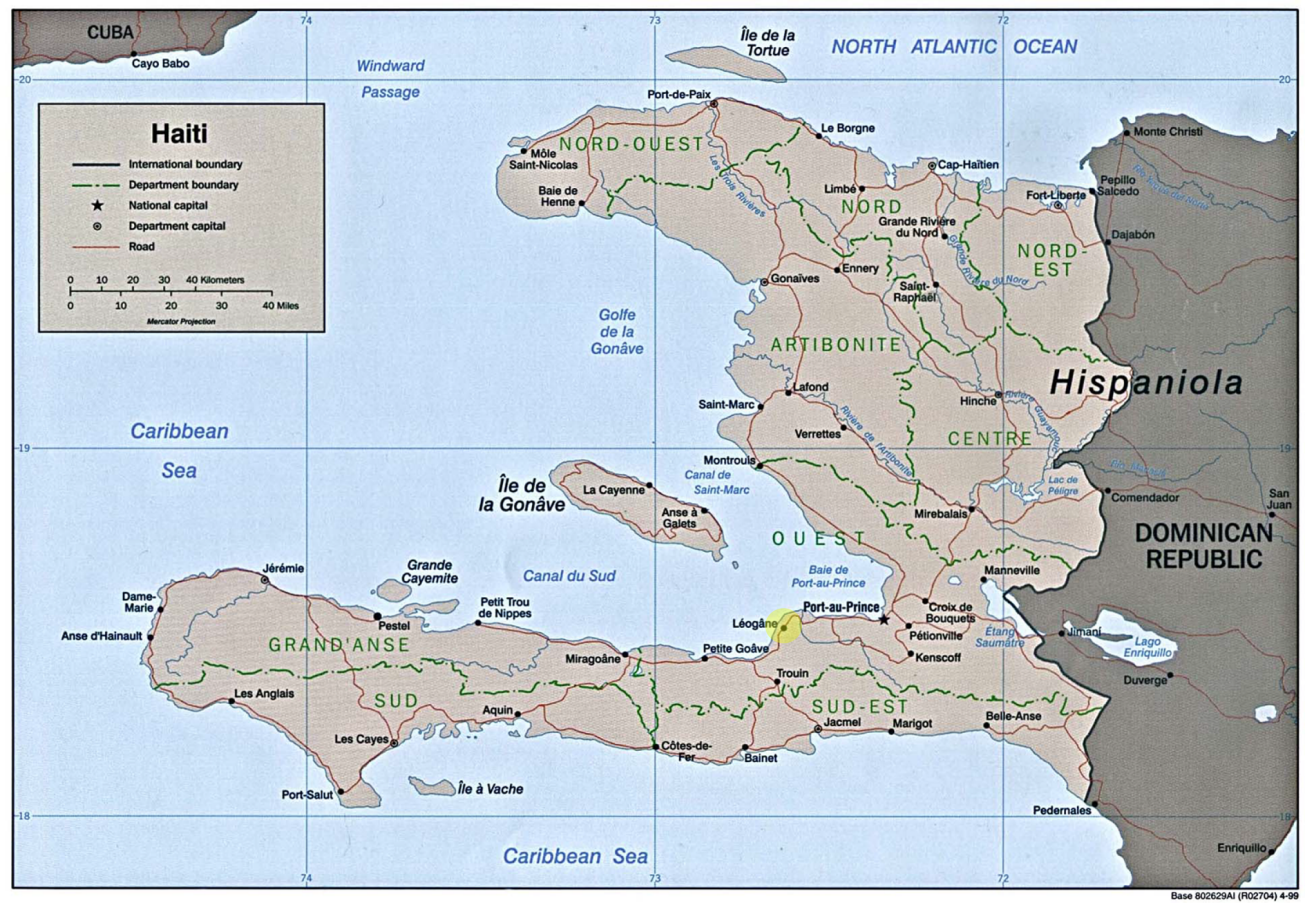
Map of Haiti, with area of Mayaro isolation highlighted. *Modified from University of Texas Perry-Castaneda Library*. http://www.lib.utexas.edu/maps/americas/haiti_rel99.pdf.

In 2016, Mayaro virus was recovered from an 8-year-old boy with an acute febrile illness in a “semirural” area (Gressier-Leogane) approximately 20 miles west of Port-au-Prince, Haiti (Fig 2) [19]. Of note, the patient was found to be coinfected with dengue virus [19]. The fact that Mayaro virus infection was found in someone from a nonforest area and it occurred in the context of a dengue coinfection suggests that *Ae*. *aegypti* may have been the mosquito vector responsible for transmission. It has been further noted that Haiti is not native to wild nonhuman primates, which could suggest a different reservoir or human-to-human transmission by *Aedes* mosquitoes.

Could Mayaro virus infection become the “next chikungunya” in the Americas? Drs. Mario Luis Garcia de Figueiredo and Luiz Tadeu Moraes Figueiredo from Brazil’s prestigious University of Sao Paulo have suggested that both of these alphaviruses can “mutate and/or adapt to new zoonotic cycles and thus acquire a higher potential for emergency” to cause significant epidemics [20]. This is an important hypothesis that requires investigation but one that will be complicated to investigate given the possible immunological cross-reactivities to these 2 alphaviruses.

The Caribbean and tropical regions of Latin America have now become high-risk areas for the emergence of Mayaro virus infection epidemics. The countries at highest risk of emergence are also resource limited and lack diagnostic capacity at the local level; therefore, it is highly likely that any Mayaro virus infections would be presumed as chikungunya virus due to the similarity of clinical symptoms, possibly allowing rapid transmission and subsequent spread throughout Central and North America to occur under our global health radar.

With locally acquired infections of both chikungunya and Zika virus now occurring in Texas and Florida in the US, we would presume these areas to also be at high risk for emergence of Mayaro virus. In May of 2016, the Texas Department of State Health Services reported on the first locally acquired case of chikungunya in Cameron County, Texas [21], while 2 years earlier, the United States Centers for Disease Control and Prevention (CDC) reported on chikungunya transmission in Florida [22]. In August 2016, Zika virus emerged in Florida with the first cases of local transmission identified in Miami, and by November, the first locally acquired case was reported from the Rio Grande Valley of Texas [23]. Therefore, we are also concerned about the emergence of Mayaro virus infection in North America, including the US.

Emerging arbovirus infections have become a “new normal” for the Americas [24], including now the continental US, which has seen dengue [25], WNV, chikungunya, and Zika [26] outbreaks over the last 15 years. As we think about public health emergency preparedness and the Global Health Security Agenda (GHSA) for 2017, we now need to add Mayaro virus infection to the growing list of emerging arbovirus diseases.
